# Efficiency of Pb, Zn, Cd, and Mn Removal from Karst Water by *Eichhornia crassipes*

**DOI:** 10.3390/ijerph17155329

**Published:** 2020-07-26

**Authors:** Jin-Mei Zhou, Zhong-Cheng Jiang, Xiao-Qun Qin, Lian-Kai Zhang, Qi-Bo Huang, Guang-Li Xu, Dionysios D. Dionysiou

**Affiliations:** 1 Institute of Karst Geology, Chinese Academy of Geological Sciences, Guilin 541004, China; jinmzhou@cug.edu.cn (J.-M.Z.); qxq@karst.ac.cn (X.-Q.Q.); zhangliankai@karst.ac.cn (L.-K.Z.); qbohuang@karst.ac.cn (Q.-B.H.); 2 Faculty of Engineering, China University of Geosciences, Wuhan 430074, China; xu1963@cug.edu.cn; 3 Key Laboratory of Karst Ecosystem and Treatment of Rocky Desertification, Ministry of Natural Resources, Guilin 541004, China; 4 Environmental Engineering and Science Program, Department of Chemical and Environmental Engineering (ChEE), University of Cincinnati, Cincinnati, OH 45221, USA; 5 Key Laboratory of Karst Dynamics, Ministry of Natural Resources, Guilin 541004, China

**Keywords:** *Eichhornia crassipes*, heavy metals, removal, morphology, cation exchange, functional groups

## Abstract

This study experimentally investigated heavy metal removal and accumulation in the aquatic plant *Eichhornia crassipes*. Pb, Zn, Cd, and Mn concentrations, plant morphology, and plant functional groups were analyzed. *Eichhornia crassipes* achieved high removal efficiency of Pb and Mn from karst water (over 79.5%), with high proportion of Pb, Zn, and Cd absorption occurring in the first eight days. The highest removal efficiencies were obtained at initial Pb, Zn, Cd, and Mn concentrations of 1 mg/L, 2 mg/L, 0.02 mg/L, and 0.2 mg/L, respectively. *Eichhornia crassipes* exhibited a high bioconcentration factor (Mn = 199,567 > Pb = 19,605 > Cd = 3403 > Zn = 1913) and a low translocation factor (<1). The roots accumulated more Pb, Zn, Cd, and Mn than the stolons and leaves due to the stronger tolerance of roots. The voids, stomas, air chambers, and airways promoted this accumulation. Pb, Cd, Zn, and Mn likely exchanged with Mg, Na, and K through the cation exchange. C≡C, C=O, SO_4_^2−^, O-H, C-H, and C-O played different roles during uptake, which led to different removal and accumulation effects.

## 1. Introduction

With the global development and increase in industrialization, mining, and anthropogenic activities, the generation of municipal sewage, heavy metal-based products, and other various wastes has led to severe heavy metal pollution in the ecological environment [1]. Specifically, karst water is easily polluted by wastewater containing lead (Pb), zinc (Zn), and cadmium (Cd) [2]; however, it is difficult to treat karst water polluted by heavy metals because of the unique double-layer hydrologic structure of the surface and underground in karst areas [3]. Moreover, karst water is impacted by multiple pollution sources including industrial, agricultural, and urban sources; thus, it is a significant challenge to remediate karst water polluted by heavy metals [3]. 

Heavy metals such as Pb, Zn, Cd, and manganese (Mn) will persist and accumulate in plants and animals after their release into the ecological system because of their stability, biological accumulation, and non-degradability; thus, they are toxic to biota and can seriously endanger human and animal health [4,5]. Such contaminants can result in renal failure and chronic anemia after exposure [6]. Heavy metal poisoning incidents have become frequent in recent years; for example, there have been many reports of bone disease due to heavy metal pollution in karst water in a lead-zinc mining area in Guangxi, southwest China [7,8,9,10,11]. 

Many treatment methods, such as ion exchange [12], polymer-based methods [13], and electrochemical methods [14], have been proposed to reduce heavy metal pollution in water. However, these methods are expensive, produce toxic chemical sludge during the treatment process, and cause secondary pollution to the environment [15]. Therefore, new eco-friendly and low-cost technologies are urgently required. Phytoremediation, which involves the removal of pollutants from water environments using plants, has become an important alternative to traditional heavy metal contamination treatments due to its environmental friendliness, low cost, and less negative impact [16,17]. Thus, phytoremediation is a universally accepted and widely applied treatment for heavy metal-contaminated water [18,19]. 

*Eichhornia crassipes*, known as the common water hyacinth, is an aquatic plant with leaves and stolons that float on the water surface. It is widely distributed throughout karst areas in southwest China due to its high productivity and adaptability to the environment. *Eichhornia crassipes* has low water quality demands and can grow well in sewage; its high tolerance to heavy metals and efficient uptake of heavy metals make it a very popular phytoremediation plant [20]. Additionally, it has one of the highest purification effects of all aquatic plants [21]. Saha et al. [22] used *Eichhornia crassipes* to restore water polluted by heavy metals and achieved a Cr (VI) removal efficiency of 99.5% from wastewater over only 15 days. Moreover, Suryandari et al. [23] achieved a Pb removal efficiency of 99.71% by *Eichhornia crassipes* that was harvested after nine days. 

Recent studies on heavy metals (Pb, Zn, Cd, and Mn) uptake by *Eichhornia crassipes* plants have typically focused on the influencing factors [24], removal rate [22,23,25], and main heavy metal absorption pathways of *Eichhornia crassipes* [23,26,27]. Furthermore, the majority of studies have employed a dead biological adsorbent [28,29], whereas living *Eichhornia crassipes* exhibits an efficient adaptation to hydroponics and capacity for heavy metal uptake at higher pollutant concentrations in the water [30]. Many previous studies have illustrated the effects of heavy metals on plants growth. The use of plants as phytoremediation materials is also well-known. This is especially the case for the heavy metal Pb and the plant *Eichhornia crassipes*. However, the specific combined remediation of Pb, Zn, Cd, and Mn has not previously been reported prior to our study, making our work an original and a novel contribution. Previous studies have demonstrated the enormous potential of living *Eichhornia crassipes* for the purification of heavy metals in water bodies, however, these studies only analyze the phenomenon of removal. This study further analyzes the cation exchange, plant organ morphology, and organic and inorganic functional groups during the toxic metal removal in karst water by living *Eichhornia crassipes*. 

As karst water is alkaline and rich in calcium, the uptake of heavy metals from karst water by aquatic organisms is typically a difficult process [31]. Therefore, this study proposes to experimentally explore the heavy metal (Pb, Zn, Cd, and Mn) removal efficiency and accumulation of living *Eichhornia crassipes* in a karst water environment. This study analyzes the removal and uptake of Pb, Zn, Cd, and Mn by living *Eichhornia crassipes* in single and multi-component heavy metal systems in karst water and provides important insights into the application of *Eichhornia crassipes* for the treatment of karst water polluted by Pb, Zn, Cd, and Mn.

## 2. Materials and Methods

### 2.1. Materials

Naturally grown *Eichhornia crassipes* plants of a similar size were collected by the artificial salvage method from the Huixian karst wetland of Guilin, China. Rotten leaves and surface dirt were removed first, and then, the tap water was used to repeatedly rinse the *Eichhornia crassipes* plants, before cleaning them with distilled water in the laboratory. The washed plants were then soaked in tap water for seven days as a starvation treatment, in order to consume inorganic carbon in the collected *Eichhornia crassipes* plants [32,33]. This was done because the naturally grown *Eichhornia crassipes* plants in the field could have absorbed inorganic carbon such as CO_2_, HCO_3_^−^, and CO_3_^2−^ through photosynthesis from the environment before they were collected. Inorganic carbon can provide a sufficient growth matrix for *Eichhornia crassipes*, improve the removal of heavy metals, and improve the photosynthesis [31]. Leaf is the main organ for photosynthesis through the stomatal structure [34]. Since the heavy metals, Pb, Zn, Cd, and Mn are toxic to biota, this study adopted a control variable method for the remediation experiments and aimed to compare the difference between removal of Pb, Zn, Cd, and Mn by *Eichhornia crassipes* in both single and multi-component heavy metal systems. This was followed by the analysis of the damage by heavy metals to plant organ morphology. Inorganic carbon may interfere with the analysis of the removal of Pb, Zn, Cd, and Mn and the leaf morphology. Therefore, inorganic carbon in the *Eichhornia crassipes* plants should be completely consumed before the remediation experiments to ensure that the plants are in the same physiological state. The contents of main elements in the collected *Eichhornia crassipes* are shown in Table 1. The experimental karst water was collected from the Mamian-Shizishan subterranean stream outlet using polyethylene barrels. The main water quality parameters in the experimental karst water are shown in Table 2.

### 2.2. Methods

Experiments were performed in the greenhouse at Huixian. The experimental containers used were polyethylene cases (Juye, Guangzhou, China) into which the following were added: 8 L of experimental water and 30.0 g of similar-sized *Eichhornia crassipes* plant material with a 1:3 mass ratio of roots to stolons and leaves [35]. *Eichhornia crassipes* was cultivated for 24 days at 25 ± 3 °C under natural light conditions. Experiments were set up in triplicate and a control group with no heavy metals was added. Deionized water was added regularly to each case to maintain a constant overall water volume and compensate for water absorption and transpiration by the plants. For the multi-component heavy metal system, heavy metals were added as follows: three concentrations of Pb, Zn, Cd, and Mn were used using appropriate amounts of the following compounds: Pb(NO_3_)_2_, ZnSO_4_·7H_2_O, CdCl_2_·5/2H_2_O, and MnCl_2_·4H_2_O. The compounds were added to the experimental case after they were fully dissolved. The low concentrations of Pb, Zn, Cd, and Mn used in the multi-component experiments were adjusted to 0.2, 2, 0.02, and 0.2 mg/L. The medium concentrations of Pb, Zn, Cd, and Mn were adjusted to 0.5, 5, 0.05, and 0.5 mg/L. The high concentrations of Pb, Zn, Cd, and Mn were adjusted to 1, 10, 0.1, and 1 mg/L. For the single heavy metal system, the respective compounds listed above were used as the source of the heavy metals, as described in previous research [25,32]. The concentrations of Pb, Zn, Cd, and Mn used in the single-component experiments were adjusted to 0.2, 2, 0.02, and 0.2 mg/L, respectively. The Pb, Zn, Cd, and Mn concentrations were set according to the polluted water in the field, such as the groundwater around lead-zinc tailings in the southwest China. Inductively Coupled Plasma Mass Spectrometry (ICP-MS), Scanning Electron Microscopy (SEM), and Fourier-Transform Infrared Spectrometry (FTIR) methods were used to analyze the removal and uptake of Pb, Zn, Cd, and Mn by *Eichhornia crassipes* in karst water, plant organ morphology, and organic and inorganic functional groups.

### 2.3. Parameters for Analysis

The analysis parameters included the concentrations of Pb, Zn, Cd, and Mn in the karst water; element contents in *Eichhornia crassipes*; wet weight, dry weight, and Scanning Electron Microscopy (SEM) images and Fourier-Transform Infrared Spectrometry (FTIR) data for the *Eichhornia crassipes* plant organs. The water samples were collected and the concentrations of Pb, Zn, Cd, and Mn in the water samples were tested every eight days using Inductively Coupled Plasma Mass Spectrometry (ICP-MS) (iCAP Q, Thermo Fisher, Waltham, MA, USA) based on GB/T 5750.6-2006 (The National Standard of the People’s Republic of China: Standard examination methods for drinking water, Metal parameters). The *Eichhornia crassipes* plants were taken out from the polyethylene case, washed with tap water and distilled water, and then divided into roots, stolons, and leaves at the end of the experiments. Both wet weight and dry weight of the *Eichhornia crassipes* plant organs were measured by the electronic balance. The contents of elements (Pb, Zn, Cd, Mn, Ca, Mg, Na, K, and Fe) in the different *Eichhornia crassipes* plant organs were tested by ICP-MS (iCAP Q, Thermo Fisher, Waltham, MA, USA) based on GB5009.268-2016 (The National Standard of the People’s Republic of China: National food safety standard, Determination of multiple elements in foods). The fresh *Eichhornia crassipes* plant organs were treated by one-step freeze-drying with tert-butanol, and then the dried plant organ morphology was scanned by SEM (JEM-6490 LV, JEOL, Akishima, Tokyo, Japan) [35,36]. The samples were prepared via the potassium bromide squash technology, and then, the organic and inorganic functional groups of *Eichhornia crassipes* involved in the absorption process of Pb, Zn, Cd, and Mn were investigated by FTIR (Spectrum TWO, Perkin Elmer, Waltham, MA, USA) [35]. The removal efficiency, bioaccumulation quantity (BAQ), bioconcentration factor (BCF), translocation factor (TF), and relative growth rate (RGR) were determined using the experimental results obtained from this study and calculated via the equations reported by Zhou et al. [35] and Lacerda et al. [37]. Graphical analysis was conducted using the Origin 9 software (OriginLab, Northampton, MA, USA). 

### 2.4. Statistical Analysis

The normality of the experimental data was first determined using the Kolmogorov–Smirnov (K–S) test via SPSS Statistics 19 (IBM, Armonk, NY, USA) and the data fitted normal distribution (*p* > 0.05). The statistical differences between experimental treatments were determined using one-way and two-way ANOVA (*p <* 0.05) after variance homogeneity was verified via SPSS Statistics 19 (IBM, Armonk, NY, USA). The Tukey test was used after one-way and two-way ANOVA. The correlation analysis of Spearman rank was used to determine the statistical correlations via SPSS Statistics 19 (IBM, Armonk, NY, USA).

## 3. Results and Discussion

### 3.1. Pb, Zn, Cd, and Mn Removal in the Single Heavy Metal System 

The removal of Pb, Zn, Cd and Mn by *Eichhornia crassipes* showed a similar trend over time (Table 3). In the first eight days, the removal efficiencies exhibited the following order: Pb (65.19%) > Cd (58.16%) > Mn (37.30%) > Zn (17.17%). In the first 16 days, the order changed to Mn (83.62%) > Pb (77.13%) > Cd (64.88%) > Zn (22.50%). After the entire culture cycle (24 days), the order of the removal efficiencies was Mn (97.69%) > Pb (85.31%) > Cd (72.83%) > Zn (31.00%). The *p*-value was 0.000 (<0.01), suggesting that the differences between the removal efficiencies of Pb, Zn, Cd, and Mn over time were very significant. Specifically, removal efficiency increased with increasing culture time. Similarly, previous studies have reported a decrease in heavy metal concentrations in the remaining aqueous solution with increasing culture time of the experiment [38,39]. Pb, Zn, and Cd removal was highest in the first eight days (65.19%, 17.17%, and 58.16%, respectively), whereas Mn removal was highest during the second eight days (46.32%). Previous research states that substantial heavy metal removal can be achieved in a short time by *Eichhornia crassipes* [23]. Moreover, *Eichhornia crassipes* can effectively remove appreciable quantities of heavy metals from fresh water, especially at low concentrations [40]. 

### 3.2. Pb, Zn, Cd, and Mn Removal in the Multi-Component Heavy Metal System 

The removal efficiencies over the entire culture cycle were as follows: Mn (92.61%) > Pb (79.50%) > Zn (64.21%) > Cd (50.00%) at low concentration (Pb: 0.2 mg/L, Zn: 2 mg/L, Cd: 0.02 mg/L, and Mn: 0.2 mg/L), Mn (91.25%) > Pb (88.00%) > Zn (44.53%) > Cd (33.33%) at medium concentration (Pb: 0.5 mg/L, Zn: 5 mg/L, Cd: 0.05 mg/L, and Mn: 0.5 mg/L), and Pb (92.69%) > Mn (88.04%) > Zn (53.06%) > Cd (44.23%) at high concentration (Pb: 1 mg/L, Zn: 10 mg/L, Cd: 0.1 mg/L, and Mn: 1 mg/L) (Table 4). 

Pb removal increased with increasing culture time at low concentration and reached a maximum on day 24. However, at both medium and high concentrations, Pb removal on day 8 was higher than that on day 16 and day 24. Mn removal reached a maximum on day 24 at both low and high concentrations and Mn removal on day 16 was higher than that on day 8 and day 24 at medium concentration. Zn and Cd removal also increased with increasing culture time. The *p*-value was 0.000 (<0.01), showing that the differences between Pb, Zn, Cd, and Mn removal in the multi-component heavy metal system at different concentrations were very significant. The removal of Pb, Zn, Cd, and Mn by *Eichhornia crassipes* was highest at the initial concentrations of 1 mg/L, 2 mg/L, 0.02 mg/L, and 0.2 mg/L, respectively. The plants exhibited a certain tolerance to heavy metals; however, they became withered, yellow, and necrotic when the concentrations of heavy metals in the water exceeded the plant’s maximum tolerated concentration to heavy metals [41,42]. Zhou et al. [43] proved that the initial concentrations of heavy metals in an aqueous solution had a significant effect on the removal efficiency of *Eichhornia crassipes*; i.e., high concentrations of heavy metals required more active absorption sites for removal. Thus, removal was low when the initial concentrations of heavy metals were low because the active sites of *Eichhornia crassipes* were not effectively utilized. The active sites became gradually occupied with an increase in the initial concentrations of heavy metals until reaching equilibrium. If additional heavy metals were added after the equilibrium stage, the concentrations of heavy metals in the solution would correspondingly increase, thus leading to a decrease of the removal efficiency [44].

In general, *Eichhornia crassipes* had a good removal effect for Pb and Mn in both single and multi-component heavy metal systems. The removal efficiencies of Pb and Mn reached over 79.50% after the entire culture cycle, whereas the maximum removal efficiencies of Zn and Cd were 64.21% and 50.00% in the multi-component heavy metal system, respectively. Comparing the removal of Pb, Zn, Cd, and Mn in the multi-component heavy metal system at the low concentration level with that in the single heavy metal system, the present study found that Zn removal was higher in the multi-component heavy metal system than that in the single heavy metal system, but Pb, Cd, and Mn removal was lower in the multi-component heavy metal system at the low concentration level. The process of removing metal ions by aquatic plants may be significantly affected by the presence of other competitive ions in the multi-component heavy metal system [45]. Zheng [45] reported that the presence of Cu could significantly inhibit the absorption of Cd but could promote the absorption of Cr by *Eichhornia crassipes*. Mahamadi and Nharingo [21] analyzed the interaction between heavy metals in the process of removing Pb, Cd, and Zn in binary and ternary systems using *Eichhornia crassipes* and found that the combined action of Pb, Cd, and Zn was antagonistic. Pb presence suppressed the removal of Zn and Cd from water, but Pb was effectively removed from water in the presence of both Zn and Cd. However, it was speculated in this study that the presence of Pb, Cd, and Mn together improved the Zn removal from water at the low concentration level of heavy metals but that the removal of Pb, Cd, and Mn was suppressed by the presence of Zn. This study used a quaternary system containing Pb, Zn, Cd, and Mn, rather than the ternary system containing Pb, Cd, and Zn. The physicochemical properties of Pb, Zn, Cd, and Mn, such as atomic weight, coordination number, magnetism, charge, electronegativity, and reduction are different [21], and the Mn presence may change the interaction effect of Pb, Zn, and Cd during the removal by *Eichhornia crassipes* from water. Moreover, the heavy metal concentrations were different in the present study and Mahamadi and Nharingo’s study. The results obtained in this study suggest that Pb, Zn, Cd, and Mn may display synergistic or antagonistic effects in the multi-component heavy metal system during removal by *Eichhornia crassipes*; therefore, this should be examined in future research.

### 3.3. Accumulation Pathways of Pb, Zn, Cd, and Mn in Eichhornia crassipes

The bioaccumulation quantity (BAQ), bioconcentration factor (BCF), and translocation factor (TF) of Pb, Zn, Cd, and Mn in the distinct *Eichhornia crassipes* plant organs, i.e., roots, leaves and stolons, are shown in Table 5. The growth of *Eichhornia crassipes* plants in karst water with Pb, Zn, Cd, and Mn, respectively, is shown in Table 6.

The bioaccumulation quantity (BAQ) is the mass of heavy metal uptake by biomass per unit mass. It is expressed as the difference between the heavy metal content in the whole plant or a certain plant organ, per unit mass, before and after the experiment [46]. In this study, the BAQ was calculated in mg/kg using the heavy metal content in the whole *Eichhornia crassipes* plant or a certain *Eichhornia crassipes* organ on day 0 and day 24 in the single component experiment. Pb, Zn, Cd, and Mn exhibited the same bioaccumulation quantity trends in the roots, stolons, and leaves of *Eichhornia crassipes*. The *p*-value was 0.028 (<0.05), suggesting the differences between the BAQ of Pb, Zn, Cd, and Mn in *Eichhornia crassipes* were significant. The BAQ showed the same order for all plant parts (Zn > Pb > Mn > Cd), which was related to the initial concentration of the heavy metals in the experimental water. The initial concentrations of Pb, Zn, Cd, and Mn in the single-component experiments were 0.2, 2, 0.02, and 0.2 mg/L, respectively. The ratio between Pb, Zn, Cd and Mn are in the same order as those in the multiple-component experiments. That is, Zn was 10 times the concentration of Pb, and Mn and 100 times the concentration of Cd, which provided the largest Zn supply amount and made the most significant contribution to the Zn bioaccumulation quantity by *Eichhornia crassipes*. Shirinpur-Valadi et al. [39] reported that metal BAQ in *Eichhornia crassipes* increased when the metal concentration in the water increased. Similar studies also reported that increasing metal concentrations led to higher BAQ during the culture time of *Eichhornia crassipes* in water [47,48]. The BAQ of Pb, Zn, Cd, and Mn in the roots was 9.99, 9.47, 3.09, and 24.48 times that those in the stolons and leaves, respectively, indicating a much higher contribution of the roots to heavy metal BAQ. Suryandari et al. [23] cultured *Eichhornia crassipes* in water with 7 mg/L Pb for 9 days and found that the Pb BAQ was higher in roots than that in leaves. A similar study also reported that the *Eichhornia crassipes* roots accumulated much more Zn than the aerial organs when *Eichhornia crassipes* was exposed to water with 0, 2, 4, 6, and 9 mg/L Zn for 24, 48, and 72 h [48]. The results obtained in the current study suggested that the root was the major organ to accumulate Pb, Zn, Cd, and Mn.

The bioconcentration factor (BCF) indicates the capacity of aquatic plants to absorb heavy metals [49]. BCF > 1 means that the plant is a heavy metal accumulator. The BCF values were as follows: Mn (199,567) > Pb (19,605) > Cd (3404) > Zn (1913), suggesting that *Eichhornia crassipes* was a potential heavy metal accumulator for Mn, Pb, Cd, and Zn. Woldemichael et al. [47] also found that *Eichhornia crassipes* exhibited high BCF when the heavy metal concentrations were low in water. The high BCF of *Eichhornia crassipes* is due to its strong adaptability to the environment and its morphological structures. The cell walls of roots are a biological semi-permeable membrane, thereby making it difficult for pollutants to penetrate; thus, pollutants accumulate in the roots [34]. Moreover, the large interface between the roots and water acts as a filter layer that enables the accumulation of heavy metals and improves the water quality [20]. The correlation between the BCF and removal rate of Pb, Zn, Cd, and Mn was significant when the confidence (bilateral) was 0.01. Both the BCF and removal efficiency showed the same trend, i.e., Mn (97.69%) > Pb (85.31%) > Cd (72.83%) > Zn (31.00%), indicating that the reduced heavy metals in the experimental water were indeed absorbed by *Eichhornia crassipes*. 

The translocation factor (TF) represents the capacity of an aquatic plant to transfer heavy metals from the underground part to the above-ground part. TF > 1 suggests that this aquatic plant is highly capable of transferring heavy metals. In this study, the TF values were all less than 1, suggesting that *Eichhornia crassipes* had a weak ability to transfer Pb, Zn, Cd, and Mn from its roots to the stolons and leaves. This is likely because *Eichhornia crassipes* protects the photosynthetic organs, such as leaves and young stolons [34]. Plants can protect their photosynthetic organs from heavy metal toxicity by partitioning the roots from the leaves and stolons, thereby accumulating poisonous heavy metals in the roots [47,50]. 

The relative growth rate (RGR) indicates plant growth changes. It is expressed in Equation (1).

(1) $$RGR=(\ln W_{2}-\ln W_{1})/\left(T_{2}-T_{1}\right),$$

where $W_{2}$ and $W_{1}$ are the dry weight and the times are $T_{2}$ and $T_{1}$ [37]. In this study, RGR was calculated using the plant dry weight on day 0 and day 24. The *p*-value was 0.000 (<0.01), showing that the differences between the RGR values of *Eichhornia crassipes* plants in karst water with Pb, Zn, Cd, and Mn were very significant. RGR values of *Eichhornia crassipes* plants on day 24 were as follows: Mn (0.0254) > Pb (0.0170) > Cd (0.0146) > Zn (0.0104). The RGR exhibited the same trends as the removal rate and BCF (Mn > Pb > Cd > Zn). Thus, both the *Eichhornia crassipes* growth and tolerance to heavy metals followed the order: Mn, Pb, Cd, and Zn. This finding was with respect to the descending order of heavy metal concentration (from high to low) in this study. The results of this study suggest that *Eichhornia crassipes* is an efficient phytoremediation material for the uptake of Pb, Zn, Cd, and Mn from karst water and can be used to restore karst water polluted by heavy metals in China and around the world.

### 3.4. Ion Exchange Analysis

According to ICP-MS analysis, the main metal elements in *Eichhornia crassipes* before Pb, Zn, Cd, and Mn uptake were Ca, Mg, Na, K, Fe, and Mn, with values ranging from 0.20% to 5.02%. The K content was the highest (5.02%) and the heavy metal content was very low (Table 7). After uptake, the contents of Pb, Zn, Cd, and Mn all increased, whereas the contents of Mg, Na, and K decreased. Therefore, a cation exchange mechanism was proposed to explain the exchange of Pb, Cd, Zn, and Mn with Mg, Na, and K during the experiments. Similar results showed copper ions and chromium ions were absorbed, whereas Ca, Mg, and K were discharged from *Eichhornia crassipes*, suggesting that Cu and Cr displaced Ca, Mg, and K [51]. This study speculated that the cation exchange occurred in both studies, although the elements that exchanged with the heavy metals were different due to the different properties of the heavy metals. In this study, the contents of Mg, Na, and K in the roots decreased significantly more than those in the stolons and leaves, indicating that cation exchange was stronger in the roots. 

### 3.5. Morphological Analysis 

**Figure 1:**
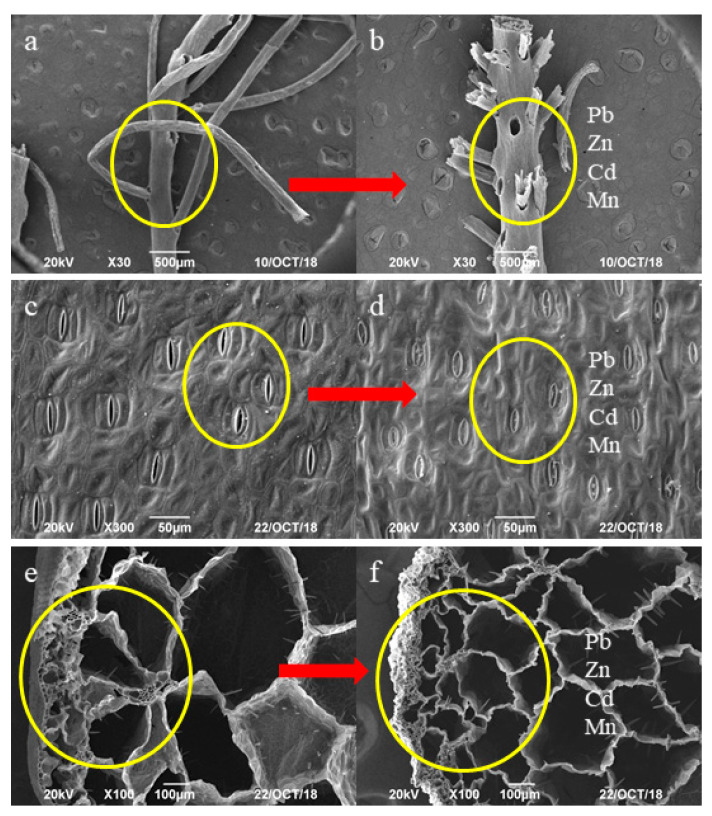
Scanning Electron Microscopy (SEM) images of (**a**) the roots, (**c**) the leaves, and (**e**) the stolons before the experiment and (**b**) the roots, (**d**) the leaves, and (**f**) the stolons in the karst water after uptake of Pb, Zn, Cd, and Mn.

The morphological features of the *Eichhornia crassipes* plants organs, i.e., roots, leaves, and stolons, were observed by SEM. Roots are important nutrient organs of *Eichhornia crassipes* during growth and are the key locations for removal of pollutants. The root system comprises taproots and fibrous roots, with voids between the two (Figure 1a) that allow ventilation and the beneficial exchange of gas and absorption of oxygen. The epidermal cells of the roots are oblong and closely arranged, and some cortical parenchyma cells form large schizogenous intercellular spaces [34]. The cell walls of the roots and their large surface area enhance the adaptability of *Eichhornia crassipes* to aquatic environments and help improve the water quality [20,34]. As shown in Figure 1b, some fibrous roots broke off after Pb, Zn, Cd, and Mn uptake; however, there was no significant change in the voids, indicating that Pb, Zn, Cd, and Mn exhibited minimal toxicity to the roots of *Eichhornia crassipes*. 

Leaves are the major photosynthetic organs during the growth and development of the *Eichhornia crassipes* plants. The leaf area is relatively large, with numerous stomas evenly distributed on the epidermis (Figure 1c). The most evident feature is the highly developed stomatal structure on the epidermis. Moreover, some sponges are organized into ventilation cavities. As shown in Figure 1d, most of the stomas on the leaves closed after Pb, Zn, Cd, and Mn uptake, suggesting that such heavy metals severely damaged the structure of the leaves and that the leaves had a weak tolerance to Pb, Zn, Cd, and Mn. Rani et al. [52] demonstrated that stomatal closure occurred after *Eichhornia crassipes* was exposed to safranin. In this study, the key effect of heavy metals in *Eichhornia crassipes* was a reduction in the number of open stomas. 

The stolons have a developed intercellular epidermis, a spongy cortex with developed aerenchyma, vascular bundles with no connection, and medulla composed of parenchyma cells in the center. Moreover, the stolons have a strong capacity for gas storage and gas exchange. Figure 1e shows that the stolons have a complicated network of systems that comprises the single vascular bundle sheath and interconnected air chambers and airways. As shown in Figure 1f, after Pb, Zn, Cd, and Mn uptake, the cortical morphology of the stolons was seriously damaged, the annular morphology composed of vascular bundles became atrophied, and the volume decreased, indicating that Pb, Zn, Cd, and Mn were harmful to the morphology of the stolons. 

The voids in the roots, stomas of the leaves, and air chambers and airways in the stolons of *Eichhornia crassipes* provided favorable conditions for the accumulation of Pb, Zn, Cd, and Mn. The morphology of the roots did not change significantly, but the morphologies of the stolons and leaves were seriously damaged after Pb, Zn, Cd, and Mn uptake, suggesting that the roots showed strong tolerance to Pb, Zn, Cd, and Mn due to a lower toxicity of these metals to the roots. These results provide strong evidence for much greater Pb, Zn, Cd, and Mn accumulation in the roots than in the stolons and leaves. The ability of *Eichhornia crassipes* to accumulate and tolerate Pb, Zn, Cd, and Mn indicates a good hyperaccumulation mechanism for the uptake of Pb, Zn, Cd, and Mn from karst water. 

### 3.6. Organic and Inorganic Functional Groups Analysis

**Figure 2:**
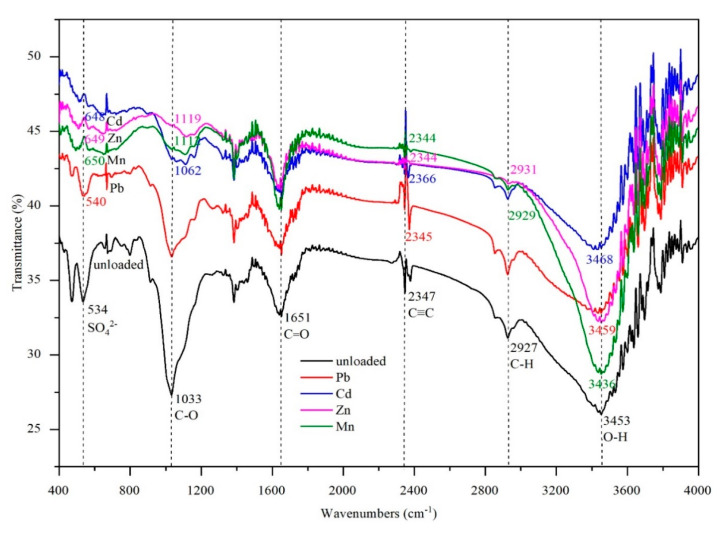
Fourier-Transform Infrared Spectrometry (FTIR) spectra of the roots of *Eichhornia crassipes* after uptake of Pb, Zn, Cd, and Mn.

The transmittance spectra for the roots of *Eichhornia crassipes* after uptake of Pb, Zn, Cd, and Mn are shown in Figure 2. The FTIR spectrum gives numerous valuable messages about the structural changes and organic and inorganic functional groups in the roots of *Eichhornia crassipes* obtained from karst water [53]. Some strong characteristic bands of functional groups were obtained from the FTIR spectra. The spectral peaks at 3453 cm^−1^, 2927 cm^−1^, 2347 cm^−1^, 1651 cm^−1^, 1033 cm^−1^, and 534 cm^−1^ represented O-H, C-H, C≡C, C=O, C-O, and the SO_4_^2^^−^ stretching vibration, respectively [44,54,55,56]. However, the organic group C=O is present in proteins, amino acids, and peptides and is often part of the structures of carbohydrates, lipids and so on. The same is true for the other mentioned organic and inorganic functional groups. The nature of the molecules interacting with heavy metals should be investigated in further studies.

As shown in Figure 2, after Pb, Zn, Cd, and Mn uptake, the shapes of the spectra were all approximately the same as those prior to heavy metal uptake, which indicated that heavy metals did not significantly change the basic chemical composition of the roots [32]. None of the C=O peaks at 1651 cm^−1^ shifted, but their intensity decreased, indicating that the structure of the cell walls had changed [57]. The strength of all SO_4_^2−^ peaks at 534 cm^−1^ decreased. However, changes in the characteristic peaks of some functional groups (C≡C, SO_4_^2−^, O-H, C-H, and C-O) exhibited differences in both position and intensity. This suggested that the functional groups played different roles when *Eichhornia crassipes* absorbed Pb, Zn, Cd, and Mn, which led to different removal and bioaccumulation effects of Pb, Zn, Cd, and Mn. Moreover, besides the different effects of C≡C, C=O, and SO_4_^2−^, O-H manifested strong functions when *Eichhornia crassipes* absorbed Pb; Zn, C-H, and C-O; Cd, O-H, and C-O; and Mn, O-H, C-O, and C-H. These differences are related to the interaction of heavy metals with the active sites (groups that can react with heavy metals) of *Eichhornia crassipes*, which resulted in new absorption bands and changes in the shape, position, and intensity of the characteristic peaks of functional groups [24]. However, desorption experiments should be performed to differentiate between exchangeable and non-exchangeable accumulated heavy metals. The fixation of heavy metals to the organic and inorganic functional groups should be analyzed in further studies.

## 4. Conclusions

This study presents novel research on the removal of Pb, Zn, Cd, and Mn from aqueous environments using biological processes and makes a significant contribution to the multi-component analysis of toxic metal removal from karst water by living *Eichhornia crassipes* plants. Specifically, this study evaluated the use of *Eichhornia crassipes* as a mitigation technique for the remediation of karst water contaminated by heavy metals. The results provide several important insights into the timing and efficiency of heavy metal removal in both single heavy metal and multi-component heavy metal systems, as well as the accumulation characteristics of different parts of the plant. Notably, living *Eichhornia crassipes* achieved high removal efficiencies with the majority of Pb, Zn, and Cd absorption occurring in the first eight days and increasing with culture time. It also exhibited a good ability to accumulate and tolerate Pb, Zn, Cd, and Mn in its roots, which accumulated more Pb, Zn, Cd, and Mn than the other plant tissues. This correlates with the evidence in our study that Pb, Zn, Cd, and Mn damaged the morphology of stolons and leaves more evidently than that of the roots. Cation exchange was proposed to explain the observed exchange of Pb, Cd, Zn, and Mn with Mg, Na, and K, and this cation exchange was stronger in the roots. C≡C, C=O, SO_4_^2−^, O-H, C-H, and C-O played different roles when *Eichhornia crassipes* absorbed Pb, Zn, Cd, and Mn. The evidence from our study strongly suggests that living *Eichhornia crassipes* may be used as an efficient phytoremediation material for the uptake of heavy metals from karst water. 

## Figures and Tables

**Table 1:** Contents of main elements in the collected *Eichhornia crassipes*.

Elements	Roots (mg/kg)	Stolons and Leaves (mg/kg)	Whole Plant (mg/kg)
Pb	7.67	0.73	2.46
Zn	98.1	38.7	53.6
Cd	0.36	0.037	0.118
Mn	1817	192	598
Ca	6600	15500	13,275
Mg	4200	1700	2325
Na	2300	830	1198
K	17,400	32800	28,950
Fe	9442	279	2570

**Table 2:** Water quality parameters in the experimental karst water.

Heavy Metal Parameters	Values (µg/L)	Regular Parameters	Values (mg/L)
Pb	<0.07	K^+^	3.46
Zn	0.88	Na^+^	1.02
Cd	<0.06	Ca^2+^	57.06
Mn	44.6	Mg^2+^	3.66
Cu	0.48	Fe^2+^	0.13
Al	38.5	Cl^−^	3.04
Cr	4.52	SO_4_^2−^	9.32
Ni	1.4	HCO_3_^−^	173.6
As	1.16	NO_3_^−^	0.84
Hg	37.8	F^−^	0.078
Co	<0.03	pH	7.25

**Table 3:** Removal efficiencies of Pb, Zn, Cd, and Mn from karst water by *Eichhornia crassipes* in the single heavy metal system (%).

Culture Time (Days)	Pb	Zn	Cd	Mn
8	65.19 ± 0.14 ^f^	17.17 ± 0.15 ^k^	58.16 ± 0.19 ^g^	37.30 ± 0.12 ^h^
16	77.13 ± 0.10 ^d^	22.50 ± 0.10 ^j^	64.88 ± 0.09 ^f^	83.62 ± 0.06 ^c^
24	85.31 ± 0.06 ^b^	31.00 ± 0.10 ^i^	72.83 ± 0.15 ^e^	97.69 ± 0.19 ^a^

Mean ± SD (*n* = 3) represents the values of three replications. The different letters mean the statistical differences between the removal efficiencies of Pb, Zn, Cd, and Mn over time are significant based on the one-way ANOVA test with culture time as a factor and the post hoc test with the Tukey test (*p* < 0.05). The same letter means no significant difference (*p* > 0.05). *p* values for comparisons between groups belonging to the same subset: ^a^ *p* = 1.000; ^b^ *p* = 1.000; ^c^ *p* = 1.000; ^d^ *p* = 1.000; ^e^ *p* = 1.000; ^f^ *p* = 0.226; ^g^ *p* = 1.000; ^h^ *p* = 1.000; ^i^ *p* = 1.000; ^j^ *p* = 1.000; ^k^ *p* = 1.000.

**Table 4:** Removal of Pb, Zn, Cd, and Mn from karst water by *Eichhornia crassipes* at different initial concentrations in the multi-component heavy metal system (%).

Concentration Level (mg/L)	Culture Time (Days)	Pb	Zn	Cd	Mn
Low concentration Pb: 0.2, Zn: 2, Cd: 0.02, Mn: 0.2	8	75.15 ± 0.16 ^j^	41.05 ± 0.13 ^q^	16.67 ± 0.22 ^z^	72.32 ± 0.13 ^k^
	16	75.50 ± 0.15 ^j^	52.78 ± 0.25 ^m^	37.50 ± 0.13 ^s^	90.64 ± 0.11 ^b,c^
	24	79.50 ± 0.23 ^i^	64.21 ± 0.20 ^l^	50.00 ± 0.18 ^n^	92.61 ± 0.09 ^a^
Medium concentration Pb: 0.5, Zn: 5, Cd: 0.05, Mn: 0.5	8	88.00 ± 0.19 ^d^	39.90 ± 0.27 ^r^	22.96 ± 0.24 ^x^	87.24 ± 0.18 ^e^
	16	86.00 ± 0.49 ^f^	42.54 ± 0.12 ^p^	31.48 ± 0.11 ^v^	91.25 ± 0.05 ^b^
	24	85.00 ± 0.37 ^g^	44.53 ± 0.15 ^o^	33.33 ± 0.11 ^t^	83.70 ± 0.10 ^h^
High concentration Pb: 1, Zn: 10, Cd: 0.1, Mn: 1	8	92.69 ± 0.10 ^a^	32.58 ± 0.07 ^u^	19.52 ± 0.12 ^y^	64.61 ± 0.10 ^l^
	16	91.10 ± 0.22 ^b^	40.67 ± 0.16 ^q^	30.77 ± 0.22 ^w^	80.06 ± 0.30 ^i^
	24	90.40 ± 0.14 ^c^	53.06 ± 0.26 ^m^	44.23 ± 0.26 ^o^	88.04 ± 0.13 ^d^

Mean ± SD (*n* = 3) represents the values of three replications. Based on the two-way ANOVA test with the concentration level and culture time as two factors and the post hoc test with the Tukey test (*p* < 0.05), the main effect of the concentration level and culture time on the Pb, Zn, Cd, and Mn removal are both significant, and the interaction between concentration level and culture time is significant. The different letters mean the statistical differences between the groups are significant. The same letter means no significant difference (*p* > 0.05). *P* values for comparisons between groups belonging to the same subset: ^a^ *p* = 1.000; ^b^ *p* = 0.098; ^c^ *p* = 1.000; ^d^ *p* = 1.000; ^e^ *p* = 1.000; ^f^ *p* = 1.000; ^g^ *p* = 1.000; ^h^ *p* = 1.000; ^i^ *p* = 0.207; ^j^ *p* = 0.963; ^k^ *p* = 1.000; ^l^ *p* = 0.853; ^m^ *p* = 0.999; ^n^ *p* = 1.000; ^o^ *p* = 0.996; ^p^ *p* = 1.000; ^q^ *p* = 0.909; ^r^ *p* = 1.000; ^s^ *p* = 1.000; ^t^ *p* = 1.000; ^u^ *p* = 1.000; ^v^ *p* = 1.000; ^w^ *p* = 1.000; ^x^ *p* = 1.000; ^y^ *p* = 1.000; ^z^ *p* = 1.000.

**Table 5:** Accumulation characteristics of Pb, Zn, Cd, and Mn in the distinct *Eichhornia crassipes* plant organs.

Plant Parts	Parameters	Pb	Zn	Cd	Mn
Roots	Bioaccumulation quantity (BAQ) (mg/kg)	1763	5252	55.94	1154
	Bioconcentration factor (BCF)	60,279	3877	10,361	643,074
Stolons and leaves	Bioaccumulation quantity (BAQ) (mg/kg)	177	1697	5.91	47.2
	Bioconcentration factor (BCF)	6024	1258	1095	51,732
Whole plant	Bioaccumulation quantity (BAQ) (mg/kg)	573	2586	18.4	324
	Bioconcentration factor (BCF)	19,605	1913	3404	199,567
	Translocation factor (TF)	0.100	0.320	0.110	0.0800

**Table 6:** Growth of *Eichhornia crassipes* plants in karst water with Pb, Zn, Cd, and Mn.

Heavy Metals	Wet Weight (g)	Dry Weight (g)	Relative Growth Rate (RGR)
Pb	45.1 ± 0.60 ^B^	2.3 ± 0.03 ^β^	0.0170 ± 0.0006 ^b^
Zn	38.5 ± 0.31 ^D^	1.9 ± 0.02 ^δ^	0.0104 ± 0.0003 ^d^
Cd	42.6 ± 0.25 ^C^	2.1 ± 0.01 ^γ^	0.0146 ± 0.0002 ^c^
Mn	55.1 ± 0.40 ^A^	2.8 ± 0.02 ^α^	0.0254 ± 0.0003 ^a^

Mean ± SD (*n* = 3) represents the values of three replications. Significant statistical differences between the experimental treatments are expressed as distinct letters based on the ANOVA test with the Tukey test (*p* < 0.05). The same letter means no significant difference (*p* > 0.05). *P* values for comparisons between groups belonging to the same subset: ^a^ *p* = 1.000, ^b^ *p* = 1.000, ^c^ *p* = 1.000, ^d^ *p* = 1.000; ^A^ *p* = 1.000, ^B^ *p* = 1.000, ^C^ *p* = 1.000, ^D^ *p* = 1.000; ^α^ *p* = 1.000, ^β^ *p* = 1.000, ^γ^ *p* = 1.000, ^δ^ *p* = 1.000.

**Table 7:** Elemental contents in *Eichhornia crassipes* (mg/kg).

Heavy Metals	Plant Parts	Ca	Mg	Na	K	Fe	Pb	Zn	Cd	Mn
Unloaded	Roots	6600	4200	2300	17,400	9442	7.67	98.1	0.360	1817
	Stolons and leaves	15,500	1700	830	32,800	279	0.730	38.7	0.0370	192
	Whole plant	13,275	2325	1198	28,950	2570	2.46	53.6	0.118	598
After Pb uptake	Roots	13,401	1157	690	3021	15,036	1771	212	0.448	2319
	Stolons and leaves	17,800	1251	663	15,301	2895	177	77.8	0.0933	302
	Whole plant	16,700	1228	670	12,231	5930	576	111	0.182	806
After Zn uptake	Roots	8614	657	696	1915	8915	6.91	5350	0.613	1676
	Stolons and leaves	19,800	851	581	6831	1067	6.65	1736	0.477	318
	Whole plant	17,004	802	610	5602	3029	6.72	2640	0.511	658
After Cd uptake	Roots	11,544	834	641	2236	10,052	62.5	104	56.3	1861
	Stolons and leaves	19,000	1293	652	17,031	1662	19.2	55.4	5.95	227
	Whole plant	17,136	1178	649	13332	3760	30.0	67.6	18.5	636
After Mn uptake	Roots	13,501	654	581	1774	5102	3.32	296	0.301	2971
	Stolons and leaves	19,400	1213	612	14,376	786	5.24	142	0.109	239
	Whole plant	17,925	1073	604	11,226	1865	4.76	180	0.157	922
